# CONNECTING MOBILITY AND SOCIAL PARTICIPATION IN OLDER ADULTS: A QUESTION OF CULTURE AND CLIMATE?

**DOI:** 10.1093/geroni/igac059.491

**Published:** 2022-12-20

**Authors:** Carl-Philipp Jansen

**Affiliations:** Robert-Bosch-Hospital Stuttgart, Stuttgart, Baden-Wurttemberg, Germany

## Abstract

The main aim of the project is to investigate the relationship between life-space mobility and social participation in older adults (65+) taking into account cultural and climatic differences across several European countries. A mixed-methods approach will be applied, combining sensor-based movement data, GPS-based geolocation data, and in-situ participant-reported information by use of experience sampling. This will allow a deeper insight into the connection of mobility and social participation taking into account different environmental and socio-cultural realities. Linkage of seasonal climate across different climate zones can help to extrapolate multi-faceted transformation of future mobility behavior of older adults under pressure of more extreme weather and climate conditions. This is of clinical relevance as there may be no “one size fits all” answer on how to best adapt to this pressure and it can be used to steer uptake and maintenance of health-enhancing mobility, social participation, and sustainable quality of life.

